# From awareness to understanding: translating mental health literacy into inclusive and empathic practice

**DOI:** 10.1192/bji.2025.10082

**Published:** 2026-02

**Authors:** Dina Aly El-Gabry

**Affiliations:** Assistant Professor of Psychiatry, College of Medicine and Health Sciences, United Arab Emirates University, Al Ain, United Arab Emirates.

**Keywords:** Mental health literacy, inclusivity, diversity, education

## Abstract

Mental health literacy (MHL) encompasses both cognitive and cultural dimensions. Low MHL sustains stigma by reinforcing misconceptions, social distancing and discrimination, discouraging help-seeking and adherence to treatment. In education, MHL extends beyond knowledge to empathy, emotional intelligence and inclusivity. Integrating MHL into psychiatry training enhances competence and compassion, cultivating openness toward mental illness. Promoting diversity and inclusion is not only a moral imperative but also vital for effective psychiatric education. Embedding MHL across learning environments fosters understanding, reduces stigma and strengthens psychiatry’s connection to the human experience in all its emotional and cultural depth.

Mental health literacy (MHL) is defined as ‘the knowledge and beliefs about mental disorders which aid their recognition, management, or prevention’.^1^ The concept has since evolved beyond knowledge and recognition to encompass the attitudes and skills that sustain well-being in everyday life. It is now recognised as a key determinant of help-seeking behaviour and mental health outcomes. Higher literacy consistently correlates with greater awareness, reduced stigma and increased use of mental health services.

Crucially, understandings and expression of mental health are shaped by culture. What one society views as distress, another may interpret as resilience or renewal. Language, traditions and shared values frame how symptoms are recognised and interpreted. MHL, therefore, cannot be understood as universal: it must reflect diverse cultural perspectives and lived experiences. Recognising this diversity is fundamental to inclusion and equitable care. Cross-disciplinary evidence also links social and cultural engagement with improved well-being.^^2^^ The World Health Organization’s World mental health report (2022) reinforces the fact that mental health promotion must be grounded in cultural understanding, community participation and equity.

## Stigma and its intersections

Stigma remains a major barrier to these aims, encompassing labelling, stereotyping, separation, status loss and discrimination. It manifests across public, personal (perceived, experienced and self-stigma) and associative forms. Experienced stigma refers to discriminatory behaviours towards individuals with mental health conditions or their associates based on negative societal beliefs.^^3^^ Low MHL perpetuates stigma by reinforcing misconceptions and stereotypes, leading to social distancing and discrimination, which discourage help-seeking and adherence to treatment, ultimately worsening outcomes.^^4^^ Strengthening MHL is therefore not merely educational but a societal imperative underpinning empathy, equity and dignity.

## Education and early literacy

In educational settings, MHL extends beyond academic attainment to social and emotional development. It equips individuals with the knowledge and confidence to manage their mental health and support others.^^5^^ Schools, colleges and universities are not only places of instruction but communities where empathy, resilience and self-awareness are cultivated. Fostering MHL in education demands more than information dissemination: it requires embedding empathy, emotional intelligence and inclusivity throughout learning.

Educators play a critical role in helping students identify strengths and sources of resilience rather than focusing solely on deficits. Reframing emotional experiences as opportunities for growth nurtures confidence, compassion and flexibility; qualities that endure beyond the classroom. Recent research links MHL to modifiable factors such as stigma towards professional help, self-efficacy, social support and positive psychological states. Addressing these determinants helps institutions build empathetic, proactive communities that inform future research, policy and practice.^^6^^

## MHL in higher education and psychiatry training

University students face high rates of depression, anxiety and sleep problems. Medical students, in particular, experience psychological distress that can impair academic performance and career progression.^^7^^ Targeted interventions grounded in MHL can enhance well-being and help-seeking in this group, with attention to equity for underrepresented or non-traditional students.^^8^^

In psychiatry training, integrating MHL strengthens professional competence and empathy. It encourages trainees to move beyond diagnostic or pharmacological frameworks to include communication, cultural sensitivity and stigma reduction. This approach fosters shared understanding, multidisciplinary collaboration and patient-centred care. Cultivating MHL within psychiatric education thus creates a culture of openness and compassion, positioning psychiatry as a discipline deeply engaged with human experience in all its emotional, social and cultural dimensions.

## Globalisation, equity and diversity

Globalisation and the internationalisation of psychiatric education highlight shared principles but also the need for local adaptation. While globalisation promotes standardised curricula, internationalisation values interchange and cultural contextualisation. Physician migration means that patients increasingly see psychiatrists from varied cultural and linguistic backgrounds, reinforcing the need for practitioners guided by self-awareness, ethical reasoning and cultural humility.^^9,10^^

Differential attainment remains a persistent concern. Systemic factors within training can create inequities based on ethnicity, language or culture.^^11^^ Addressing these inequities fosters fairness and prepares psychiatrists to deliver culturally responsive care. From both professional and personal standpoints, as psychiatrists and parents, we observe how labelling shapes identity and behaviour. When learners or children internalise negative labels, their confidence and motivation erode. Challenging such biases within supervision and training cultivates environments that support competence, compassion and growth.

## Diversity as a core educational value

Diversity and inclusion are not only moral imperatives but are essential to the delivery of effective psychiatric training. Exposure to varied cultural and clinical perspectives enriches learning. Embedding diversity, equity and inclusion within curricula enhances empathy, cultural competence and equitable care.^^12^^ Implementing these principles through mentorship, reflective supervision and intercultural training fosters institutional cultures rooted in respect and fairness, shaping psychiatrists who are clinically skilled, self-aware and globally minded.

## Cultural humility in global psychiatry

This issue of *BJPsych International* brings together studies illustrating how MHL is shaped across cultures, professions and communities. From teachers’ recognition of anxiety and depression to students’ perceptions of psychiatry, residents’ experiences with motivational interviewing and community sensitisation initiatives in Uganda and Palestine, each contribution highlights the interplay between awareness, empathy and education. Collectively, they show that literacy in mental health transcends knowledge: it is a dialogue between science, culture and human connection.

Diversity and inclusion are not only moral imperatives but are essential to the delivery of effective psychiatric training. Challenges arise when Western frameworks are applied uncritically across contexts.^^13^^ Because societies differ in how they interpret and express distress, unexamined global health efforts risk exporting assumptions that fail to resonate locally. Social determinants of mental health are shaped by community, religion and systems of meaning. Neglecting these factors can lead to misdiagnosis, ineffective treatment and the erosion of indigenous strengths. Integrating cultural context into psychiatric care is therefore vital to ensure that services remain ethical, effective and inclusive for both patients and practitioners.

## Data Availability

Data availability is not applicable to this article as no new data were created or analysed in this study.
